# Cellular basis of differential endochondral growth in Lake Malawi cichlids

**DOI:** 10.1002/dvdy.529

**Published:** 2022-08-30

**Authors:** Savannah Johnson, Brian Heubel, Carson Bredesen, Thomas Schilling, Pierre Le Pabic

**Affiliations:** ^1^ Department of Biology and Marine Biology University of North Carolina Wilmington Wilmington North Carolina USA; ^2^ Department of Developmental and Cell Biology University of California Irvine Irvine California USA

**Keywords:** cartilage, chondrocyte, craniofacial, hypertrophy, skeleton, teleosts

## Abstract

**Background:**

The shape and size of skeletal elements is determined by embryonic patterning mechanisms as well as localized growth and remodeling during post‐embryonic development. Differential growth between endochondral growth plates underlies many aspects of morphological diversity in tetrapods but has not been investigated in ray‐finned fishes. We examined endochondral growth rates in the craniofacial skeletons of two cichlid species from Lake Malawi that acquire species‐specific morphological differences during postembryonic development and quantified cellular mechanisms underlying differential growth both within and between species.

**Results:**

Cichlid endochondral growth rates vary greatly (50%‐60%) between different growth zones within a species, between different stages for the same growth zone, and between homologous growth zones in different species. Differences in cell proliferation and/or cell enlargement underlie much of this differential growth, albeit in different proportions. Strikingly, differences in extracellular matrix production do not correlate with growth rate differences.

**Conclusions:**

Differential endochondral growth drives many aspects of craniofacial morphological diversity in cichlids. Cellular proliferation and enlargement, but not extracellular matrix deposition, underlie this differential growth and this appears conserved in Osteichthyes. Cell enlargement is observed in some but not all cichlid growth zones and the degree to which it occurs resembles slower growing mammalian growth plates.

## Key Findings

- Growth rates vary greatly between cichlid endochondral growth zones.
- Differences in growth rate of the same growth zone are detected between stages and species, and between different growth zones within species.
- Cell proliferation is the main contributor to endochondral growth and its variation in cichlids.
- Unlike in tetrapods, cell enlargement is only detected in some cichlid growth zones.

## 1 INTRODUCTION

Evolutionary plasticity in skeletal development has played key roles in the morphological diversification and adaptability of vertebrates. This includes changes in embryonic patterning mechanisms as well as localized growth and remodeling during post‐embryonic development to determine the size and shape of skeletal elements. Endochondral growth zones (GZs) mediate the axial growth of cartilage‐derived bones during post‐embryonic development.^1^, ^2^ GZs include growth plates (GPs), which are well known to regulate tetrapod long bone length through differential growth, particularly in limbs.^3^ Differential growth refers to distinct growth rates either of the same GZ at different developmental stages or between species, as well as distinct growth rates between GZs in different bones.^3^, ^4^ While the cellular mechanisms underlying differential growth have received much attention in tetrapod long bones, they remain completely unexplored in ray‐finned fishes, which represent half of all living vertebrate species.

Three cellular processes contribute to the axial growth of mammalian endochondral bones: proliferation, extracellular matrix (ECM) production and cell enlargement. These processes occur at GZs through a differentiation cascade in which cartilage cells progress sequentially through three regions: resting zone (RZ), proliferative zone (PZ) and hypertrophic zone (HZ).^5^, ^6^ RZ cells divide slowly and serve as stem‐cells for the GZ. Cartilage cells divide at a higher rate in the PZ until they enter the HZ, where they differentiate into hypertrophic chondrocytes that do not divide and enlarge before dying at the chondro‐osseous junction. In rat growth plates, cell proliferation, ECM production and cell enlargement contribute 7%‐10%, 32%‐49% and 44%‐59% to axial growth, respectively.^7^ The contribution of proliferation to growth is thus relatively minor and serves to replace hypertrophic chondrocytes as they are lost at the chondro‐osseous junction.

In mammals, distinct degrees of cell proliferation, ECM production and hypertrophic cell enlargement underlie differential growth (ie, variations in growth rate). Notably, large increases in the extent of cellular enlargement in the HZ are the major drivers of accelerated growth rates in dramatic cases of limb elongation. For example, the spectacular elongation rates achieved by bat metacarpals and jerboa metatarsals result largely from exceptional degrees of cellular enlargement in the HZ: 70‐fold and 40‐fold in cell volume, respectively,^8^, ^9^ against 10‐fold in the rat proximal tibia HZ.^7^ In non‐mammalian tetrapods, such as birds and amphibians, cell proliferation, ECM production and cell enlargement contribute to axial growth of endochondral bones, but differences in cell enlargement have not been associated with differential growth between species.^10^

In ray‐finned fishes (Actinopterygians), mechanisms of differential growth at GZs remain unexplored. Teleost GZs were first described at the epiphyses of branchial bones of the gills, which resemble tetrapod long bones, except for their lack of secondary ossification centers.^11^, ^12^ Similar to tetrapods, HZ chondrocytes in zebrafish can become osteocytes and blood vessels invade the bone marrow cavity, but the marrow is fat‐filled rather than a site of hematopoiesis.^13^ In GZs of the zebrafish skull, hypertrophic chondrocyte enlargement and ECM production do not significantly contribute to axial elongation, suggesting that cell proliferation drives endochondral growth.^14^

Studies in closely related yet morphologically divergent species of East African cichlids and damselfishes (Pomacentridae family) suggest that differences in morphology appear during postembryonic development.^15^, ^16^, ^17^

Here, we describe bone growth and cell proliferation in GZs of two closely related cichlids from Lake Malawi with dramatic differences in craniofacial morphology, *Copadichromis azureus* (CA) and *Dimidiochromis compressiceps* (DC). We focus on synchondroses of the skull in four bones, the quadrate (QA), metapterygoid (MP), symplectic (SY) and hyomandibular (HM) each of which contains bidirectional GZs. We demonstrate differential growth across stages between different GZs within CA or DC, as well as differential growth within the same GZ between species. We then compare the cellular processes underlying this differential growth and show changes in cell cycle length, proliferating cell pool size and cell enlargement that account for differences in GZ performance.

## 2 RESULTS

### 2.1 Endochondral growth zones of the cichlid suspensorium

**FIGURE 1:**
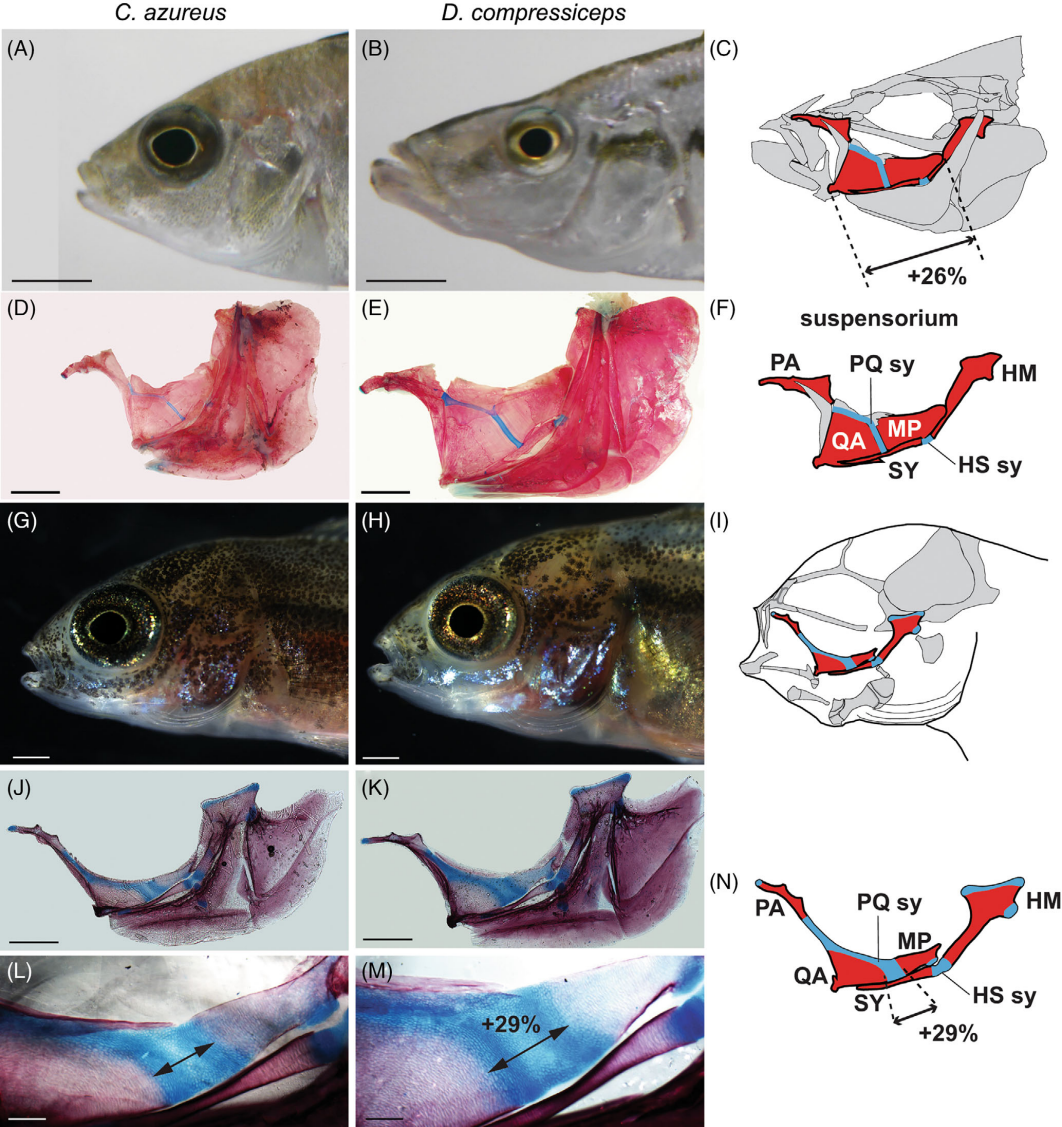
Adult bone size parallels larval synchondrosis size in the cichlid suspensorium. A‐C: Adult head morphology in *Copadichromis azureus* (CA, A) and *Dimidiochromis compressiceps* (DC, B), and underlying skeletal morphology in DC (C). (C) Adult suspensorium size is 26% greater in DC than in CA as measured between the jaw joint and the posterior‐most MP. Scale bar: 0.5 cm. D‐F: Adult CA (D) and DC (E) suspensorium skeletons and bone nomenclature (F). Bone stained red, cartilage stained blue. Scale bar: 0.5 cm. G‐I: Larval head morphology in CA (G) and DC (H), and underlying skeletal morphology (I). Scale bar: 0.5 mm. J‐N: Larval CA (J, L) and DC (K, M) suspensorium skeletons and bone nomenclature (N). Double arrows indicate PQ synchondrosis width in CA (L) and DC (M). PQ synchondrosis width is 29% greater in DC than in CA at 9CFRE stage. Bone stained red, cartilage stained blue. Scale bars: 0.5 mm (J, K) and 0.1 mm (L, M). Anterior is to the left and dorsal to the top in all figure panels. Panels D‐E are reproduced from^17^ under the Creative Commons Attribution License 4.0. Abbreviations: HM, hyomandibular; HS sy, hyosymplectic synchondrosis; MP, metapterygoid; PA, palatine; PQ sy, palatoquadrate synchondrosis; QA, quadrate; SY, symplectic.

We previously showed that the dramatic difference in head length between adult CA and DC is associated with size differences of the suspensorium.^17^ The suspensorium is a shared set of bones of the craniofacial skeleton in ray‐finned fishes that connects the jaws, opercular bones and branchial basket to the neurocranium. It derives from the first two embryonic pharyngeal arches, mandibular and hyoid (Figure 1).^18^ Here we focus on the palatoquadrate (PQ) synchondrosis as a putative GZ for the quadrate (QA) and metapterygoid (MP) bones, and on the hyosymplectic (HS) synchondrosis as a putative GZ for the symplectic (SY) and hyomandibular (HM) bones (Figure 1F). In adults of same standard length, the suspensorium is 26% longer along the anterior‐posterior axis in DC than CA as measured between the jaw‐joint and the posterior end of the MP (Figure 1A‐F).^17^ However, these bones are much more similar in size in larvae at 15 days post‐fertilization (dpf), suggesting that much of the difference in length results from later growth at GZs in juveniles (Figure 1G‐N).^17^ In addition, a 29% wider PQ synchondrosis in DC larvae than CA suggests that this GZ produces much of the skeletal size difference seen in adults as a result of more proliferation (Figure 1G‐N).^17^

**FIGURE 2:**
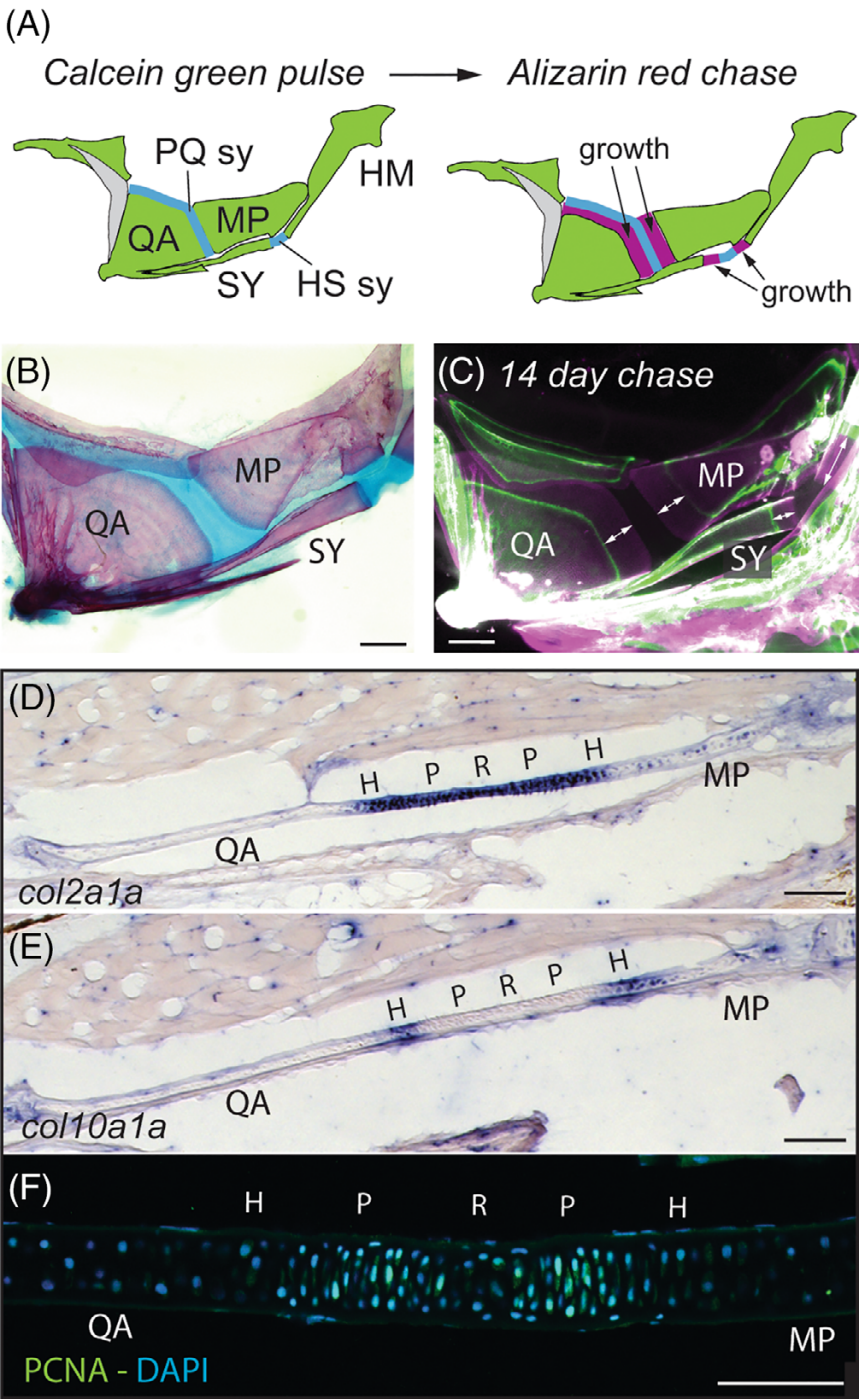
The cichlid palatoquadrate synchondrosis is a bidirectional endochondral growth zone. A: Pulse‐chase illustration: bone is first stained green by calcein green pulse (left), followed by a red stain during alizarin red chase (right). Bone deposited between pulse and chase is stained red alone. B: Alizarin red and alcian blue stain of 1.4 cm (TrL) CA suspensorium bones in red and cartilage in blue. Scale bar: 0.25 mm. C: Suspensorium of CA individual pulsed with calcein green at 1 cm (TrL) and chased with alizarin red 14 days later. Bone deposited between pulse and chase is stained in red alone and indicated by double arrows. Scale bar: 0.25 mm. D‐E: In situ hybridization for *col2a1a* (D) and *col10a1a* (E) on serial frontal cryosections of CA suspensorium. (D) *Col2a1a* labels undifferentiated chondrocytes of the resting and proliferative zones of the PQ synchondrosis. (E) *Col10a1a* labels prehypertrophic chondrocytes. Scale bar: 75 μm. F: Proliferating cell immunostaining in PQ synchondrosis with anti‐PCNA (green) antibody, DAPI stains nuclei blue. Dense PCNA‐label identifies proliferative zone chondrocytes. Scale bar: 75 μm. Anterior is to the left and dorsal to the top in all figure panels. Abbreviations: H, hypertrophic zone; HM, hyomandibular; HS sy, hyosymplectic synchondrosis; MP, metapterygoid; P, proliferative zone; PQ sy, palatoquadrate synchondrosis; QA, quadrate; R, resting zone; SY, symplectic.

To assay bone growth at the PQ and SY synchondroses, we used a pulse‐chase, live staining assay in which CA individuals (trunk length, TrL = 1.4 cm) were first stained with calcein green followed by alizarin red two weeks later (Figure 2A). Using this method, Alizarin alone stains any bone deposited after removing the calcein green. We observed localized growths of the QA and MP across the PQ synchondrosis as well as growths of the SY and HM across the HS synchondrosis (Figure 2B,C). These localized growths at the interface between synchondrosis cartilage and calcein‐stained bone support the roles of these synchondroses as GZs.

Next, we used molecular markers to further explore similarities between tetrapod and teleost GZs. Nested patterns of gene expression that mark specific sets of chondrocytes within tetrapod GZs include *type II collagen alpha a* (*col2a1a*) in RZs and PZs and *type X collagen alpha a* (*col10a1a*) in HZs. In serial cryosections of the QA synchondrosis in CA, *col2a1a* expression was detected in a central area that we identify as a RZ, flanked on each side by *col2a1a +* putative PZs (Figure 2D). On either side of this region of *col2a1a* expression we found partially overlapping domains of *col10a1a* expression, which we interpret as HZs (Figure 2E). This partial overlap is consistent with other studies in zebrafish and tetrapods.^14^, ^19^ To test if our putative *col2a1a +* PZs are proliferative, anti‐PCNA immunolabelling was used to label proliferative cells in the QA synchondrosis (Figure 2F): and this densely stained two clusters of cells flanking a less stained putative RZ, distinct from putative HZ cells in both the QA and MP (Figure 2F). These results show that suspensorium synchondroses in cichlids are GZs and highlight similarities between cichlid and tetrapod GZs.

### 2.2 Inter‐ and intra‐specific differences in endochondral growth rates

**FIGURE 3:**
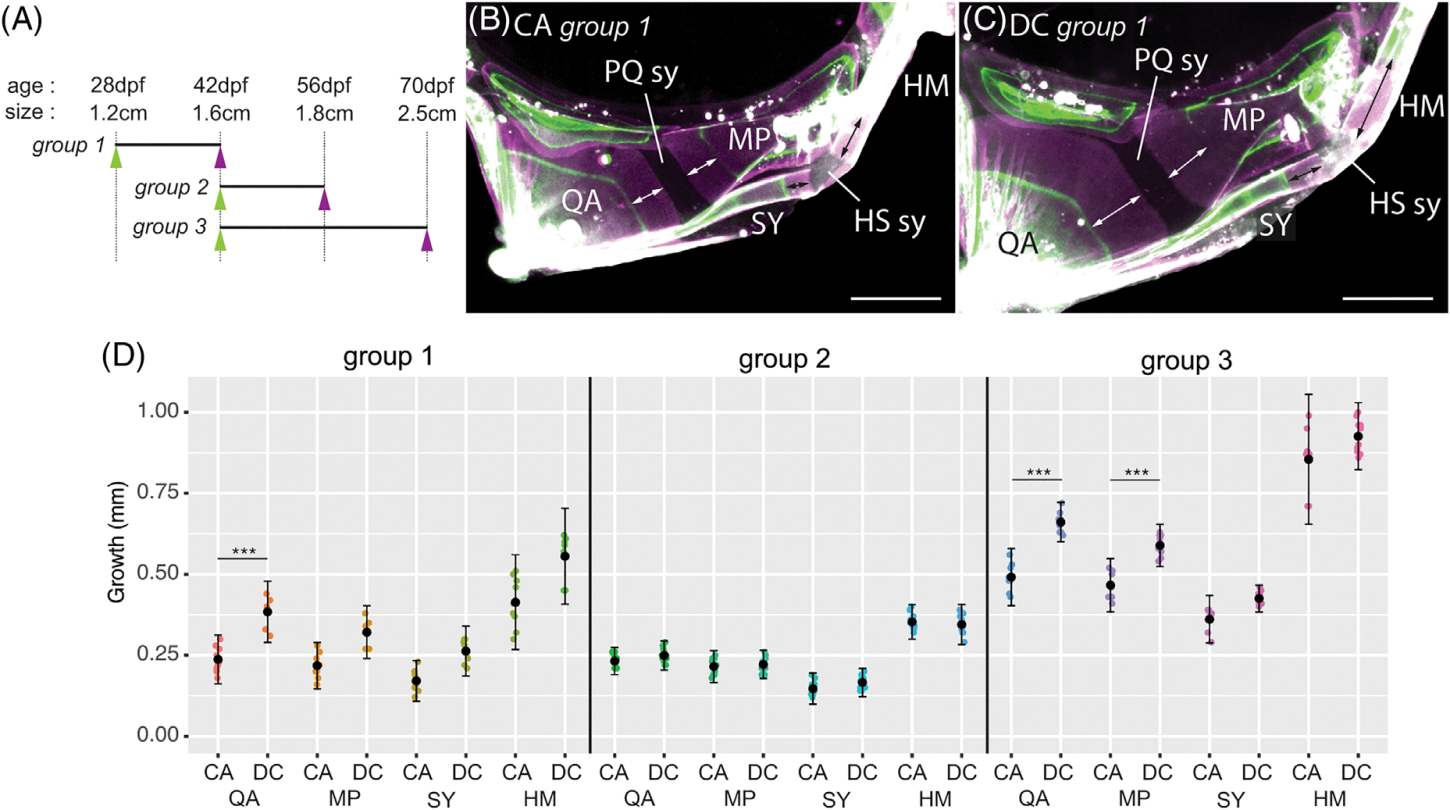
Differential growth of suspensorium bones within and between species and stages. A: Calcein‐green pulse and alizarin red chase time‐tables for groups 1, 2 and 3. B‐C: Representative results of group 1 pulse‐chase in CA (B) and DC (C). Bone deposited between pulse and chase is stained in red alone and indicated by double arrows. D: Bone growth assayed by pulse‐chase stain in groups 1‐3. (***) indicates statistical significance (*P* < 0.001, THSD test). Anterior is to the left and dorsal to the top in all figure panels. Abbreviations: HM, hyomandibular; HS sy, hyosymplectic synchondrosis; MP, metapterygoid; PQ sy, palatoquadrate synchondrosis; QA, quadrate; SY, symplectic. Scale bar: 0.25 mm

To determine if differences in GZ activity may underlie divergent adult skull morphologies, we quantified growth rates of the QA, MP, SY and HM in CA siblings and DC siblings raised together. As described above, we used a pulse‐chase live stain assay in which individuals were first stained with calcein green followed by alizarin red either two (groups 1 and 2) or 4 weeks later (group 3). New bone growth was then quantified on both sides of each synchondrosis (Figure 3).

A first pulse was conducted on 28 dpf juveniles (group 1, TrL = 1.2 cm, *n* = 5 CA and *n* = 5 DC), followed by a chase 2 weeks later (42 dpf, TrL = 1.6 cm). More growth was observed in DC than CA for each bone (QA, MP, SY, HM), and one bone in particular, the HM, grew much more than others in both species (Figure 3D and Table 1). Despite the apparent growth differences between species, only the differences in QA bone growth were statistically significant (62%, Tukey's Honest Significant Difference Test, THSD, *P*‐value = .0009, Figure 3D and Table 1). Group 1 growth rates ranged from 12 μm/day (CA SY) to 39 μm/day (DC HM, Table 1). These results show that growth rates differ by anatomical location within each cichlid species and that growth rates of at least some homologous GZs differ between species.

**TABLE 1:** Growth parameters of suspensorium bones at three stages of cichlid postembryonic development

	QA	MP	SY	HM
Group 1:1.2‐1.6 cm				
CA growth (mm)	0.24	0.22	0.17	0.42
CA growth rate (μm/day)	17.0	15.7	12.1	30.0
DC growth (mm)	0.38	0.32	0.26	0.55
DC growth rate (μm/day)	27.4	22.9	18.6	39.3
DC/CA	1.62	1.45	1.53	1.31
*P* value	.0009055	.0460438	.0896135	.0022669
Group 2:1.6–1.8 cm				
CA growth (mm)	0.23	0.21	0.15	0.35
CA growth rate (μm/day)	16.4	15.0	10.7	25.0
DC growth (mm)	0.25	0.22	0.17	0.34
DC growth rate (μm/day)	17.9	15.7	12.1	24.3
DC/CA	1.09	1.05	1.13	0.97
*P* value	.956248	.9995432	.9514136	.9989856
Group 3:1.6‐2.5 cm				
CA growth (mm)	0.49	0.47	0.36	0.85
CA growth rate (μm/day)	17.5	16.8	12.9	30.4
DC growth (mm)	0.66	0.59	0.43	0.93
DC growth rate (μm/day)	23.6	21.1	15.4	33.2
DC/CA	1.35	1.26	1.19	1.09
*P* value	.0003836	.0167578	.6394213	.3416575

To determine if GZ growth rates vary over time in the cichlid skull, a second pulse was conducted on 42 dpf CA siblings and DC siblings (TrL = 1.6 cm, *n* = 10 CA and *n* = 10 DC) raised in a common tank, followed either by a chase 14 days later (group 2, 56 dpf, TrL = 1.8 cm, *n* = 5 CA and *n* = 5 DC) or 28 days later (group 3, 70 dpf, TrL = 2.5 cm, *n* = 5 CA and *n* = 5 DC, Figure 3A). Interestingly, growth rates of CA bones from group 2 were virtually the same as those from group 1 (Figure 3E and Table 1), while DC growth rates for group 2 were lower than DC group 1 and closely matched those of CA group 2 (Figure 3E and Table 1). In contrast, DC growth rates were greater than CA's for the QA (35%, *P* value = .0004) and MP (26%, *P* value = .02) in group 3 (THSD), while growth of the SY/HM were not significantly different between the two species (Figure 3F and Table 1). These results demonstrate intraspecific growth rate changes at particular GZs during juvenile development.

### 2.3 Hypertrophy and ECM production in cichlid growth zones

In mammals, variation in HZ cell enlargement is the greatest contributor to differential endochondral growth, followed in order of importance by ECM production, and cell proliferation (Farnum et al.^8^; Cooper et al.^9^). In ray‐finned fishes the cellular basis of endochondral growth has only been examined in the zebrafish (*Danio rerio*, order Cypriniformes), where cell proliferation seems to be the main driver of growth, since HZ cell enlargement and ECM production are negligible.^14^

**FIGURE 4:**
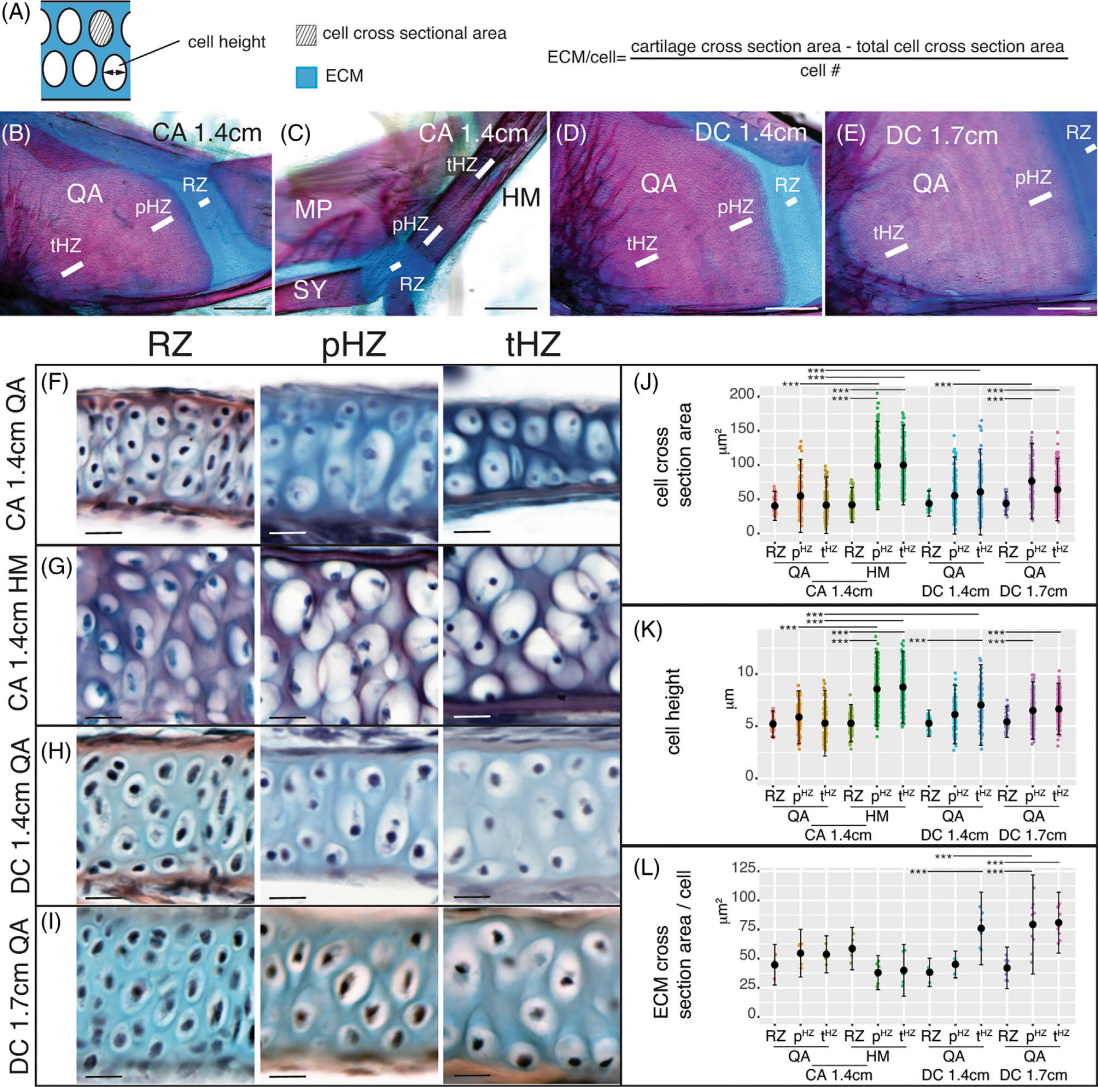
Quantification of chondrocyte size and ECM content in DC and CA endochondral growth zones. A: Chondrocyte parameters measured on histological sections. B‐E: QA (B, D‐E) and HM (C) section planes and areas shown in histological sections below. Scale bar: 0.25 mm. F‐I: histological sections through the RZ, pHZ and tHZ of the CA QA (F) and HM (G) at 1.4 cm (TrL), and the DC QA at 1.4 cm (H) and 1.7 cm (I, TrL). Scale bar: 10 μm. J‐L: cell parameter quantification in the RZ, pHZ and tHZ of the CA QA and HM at 1.4 cm (TrL), and the DC QA at 1.4 cm and 1.7 cm (TrL). (J) cell cross‐sectional area quantification. (K) cell height quantification. (L) ECM cross‐sectional area per cell. (***) indicates statistical significance (*P* < 0.001, THSD test). Abbreviations: ECM, extracellular matrix; HM, hyomandibular; MP, metapterygoid; pHZ, prehypertrophic zone; QA, quadrate; RZ, resting zone; SY, symplectic; tHZ, terminal hypertrophic zone.

To determine if cell enlargement and ECM production contribute to endochondral growth and its variation in cichlid fishes, we quantified cell cross‐sectional area, cell height and ECM/cell (ECM cross‐sectional area associated with each cell) on histological sections cut along the plane of endochondral growth in the HZ of CA and DC bones with different growth rates (Figure 4A‐E). Comparisons were made for the three cases in which we observed differential growth: (1) growth rate differences between distinct bones (QA and HM) within one species (CA) at a particular stage (Figure 4B,C), (2) growth rate differences between homologous bones (QA) of distinct species (CA and DC) at the same stage (Figure 4B,D), and stage‐dependent variation (1.4 cm vs. 1.7 cm TrL) in growth rates of a particular bone (QA) within one species (DC, Figure 4D,E).

QA and HM bones of 1.4 cm (TrL) CAs (*n* = 3) were examined because growth rate is greater for the HM than for the QA during the 28‐42 dpf period (1.2‐1.6 cm TrL; Table 1). Cell and ECM parameters were quantified in CA at the RZ and two regions of the HZ to detect any changes arising during cell maturation: the prehypertrophic zone (pHZ) located directly next to the PZ and the terminal HZ (tHZ) located next to the chondro‐osseous junction (Figure 4B,C,F,G). In the QA, cell size and ECM parameters were constant across regions (Figure 4F,J‐L and Table 2). In the HM, cell cross‐sectional area and height showed significant increases: 2.4‐fold and 1.6‐fold respectively, between the RZ and pHZ/tHZ zones (*P* < .001 THSD; Figure 4G,J‐L and Table 2), while ECM/cell did not change. These observations suggest that cell enlargement in the pHZ likely contributes to growth in the CA HM, but not the QA. We estimate HM cell enlargement at 3.3‐fold between RZ (177.5 fL) and pHZ (584.9 fL), using average cell radii and the ellipsoid volume formula (Table 2).

**TABLE 2:** Quantification of cell size and ECM amount in endochondral growth zones of CA and DC

	/pHZ QA CA 1.4cm	*P* value	/pHZ QA DC 1.7 cm	*P* value
pHZ HM CA 1.4 cm				
Cross‐sectional area (μm^2^)	1.81	0***		
Cell height (μm)	1.46	0***		
ECM cross‐sectional area/cell (μm^2^)	0.70	.488		
pHZ QA DC 1.4 cm				
Cross‐sectional area (μm^2^)	1.01	1	0.73	0***
Cell height (μm)	1.03	.983	0.94	.666
ECM cross‐sectional area/cell (μm^2^)	0.83	.998	0.57	.0009***

	/tHZ QA CA 1.4 cm	*P* value	/tHZ QA DC 1.7 cm	*P* value
tHZ HM CA 1.4 cm				
Cross‐sectional area (μm^2^)	2.4	0***		
Cell height (μm)	1.6	0***		
ECM cross‐sectional area/cell (μm^2^)	0.8	.993		
tHZ QA DC 1.4 cm				
Cross‐sectional area (μm^2^)	1.5	0***	0.9	.989
Cell height (μm)	1.3	0***	1.1	.633
ECM cross‐sectional area/cell (μm^2^)	1.6	.023	0.9	.997

QA bones of CA and DC (1.4 cm TrL; *n* = 3) at the same stage were compared because the QA growth rate is greater in DC than in CA during the 28‐42 dpf period (1.2‐1.6 cm TrL; Table 1). Unlike in CA, cell height and ECM/cell increased 1.3 and 1.5‐fold, respectively, between the DC RZ and tHZ zones (*P* < .001 THSD; Figure 4H,J‐L and Table 2). Compared to CA values, tHZ cell cross‐sectional area and height were 1.5 and 1.3‐fold greater in DC, respectively (*P* < .001 THSD; Figure 4H,J‐L and Table 2), suggesting that differences in cell enlargement as cells mature in the HZ may underlie QA growth rate differences between CA and DC.

Lastly, DC QA bones of different stages, 1.4 cm and 1.7 cm (TrL; *n* = 3), were compared because the DC QA growth rate is greater during the 28‐42 dpf period (1.2‐1.6 cm TrL) than during the 42‐56 dpf period (1.6‐1.8 cm TrL; Table 1). In 1.7 cm (TrL) DC, significant increases in cell cross‐sectional area (1.7‐fold), cell height (1.2‐fold) and ECM/cell (1.9‐fold), were found between the RZ and pHZ, without further increase in the tHZ (*P* < .001 THSD; Figure 4I,J‐L and Table 2). In comparison to 1.4 cm (TrL) values, 1.7 cm(TrL) cell cross‐sectional area was greater (1.4‐fold) in the pHZ, and ECM/cell was greater (1.8‐fold) in the pHZ (*P* < .001 THSD; Figure 4I,J‐L and Table 2). However, these value increases do not positively correlate with the lower QA growth rate observed at 1.7 cm (TrL).

In summary, we observe a positive correlation between the degree of cell enlargement and growth rate in cichlid GZs but not ECM deposition, suggesting that variations in cell enlargement may underlie growth rate differences.

### 2.4 Differences in proliferative cell number and cell cycle length underlie interspecific and intraspecific growth rate differences

Chondrocyte proliferation contributes very little to endochondral growth and its modulation within and between mammalian species.^3^, ^7^ In contrast, in cichlids, cell enlargement and increases in ECM/cell are negligible in GZs, suggesting that cellular proliferation plays a more important role in endochondral growth.

**FIGURE 5:**
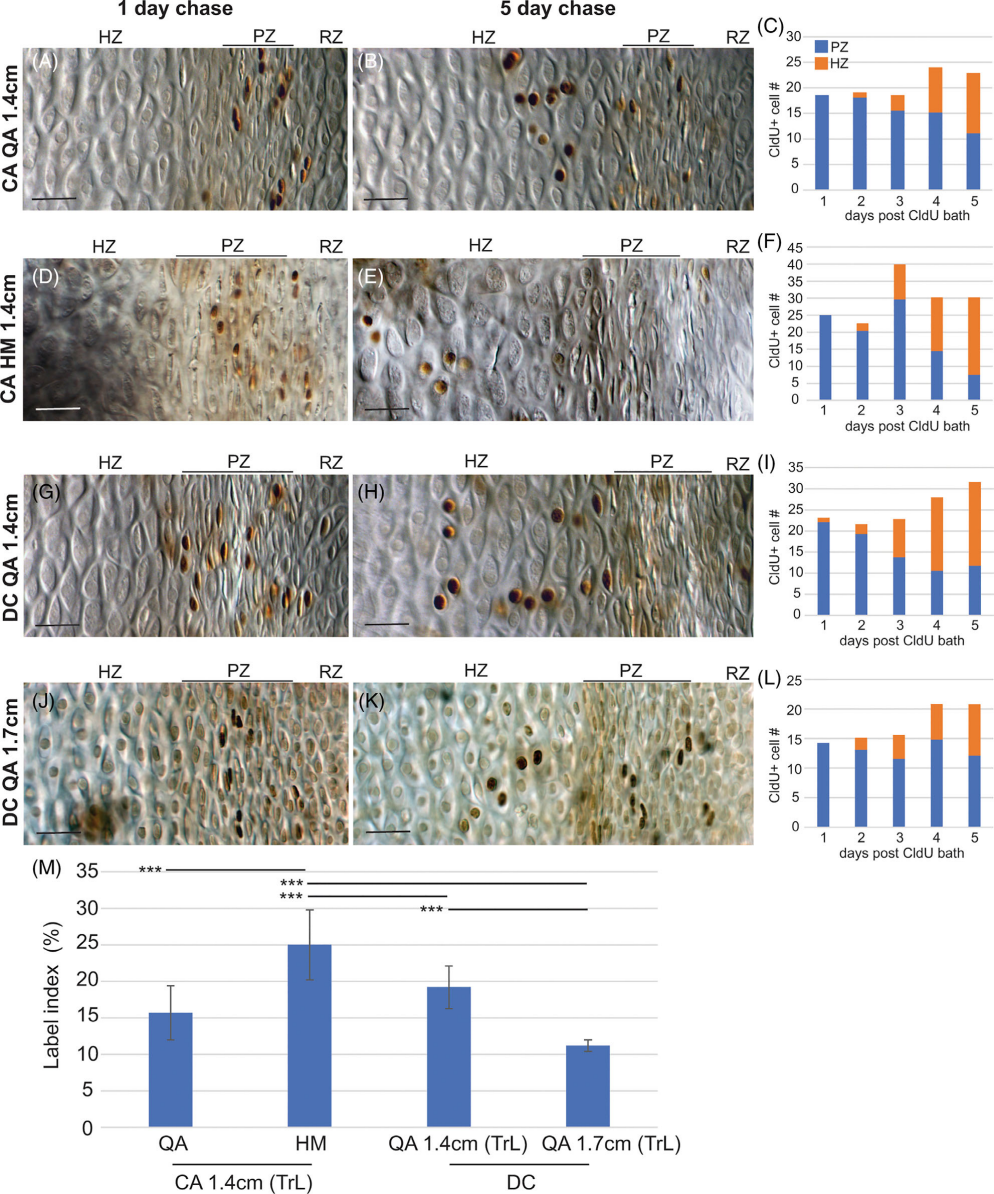
Quantification of chondrocyte proliferation in DC and CA endochondral growth zones. A‐L: Anti‐CldU immunostaining of CldU‐labeled cells in: QA of CA 1.4 cm (Trunk Length, TrL, A‐C), HM of CA 1.4 cm (TrL, D‐F), QA of DC 1.4 cm (TrL, G‐I) and QA of DC 1.7 cm (TrL, J‐L). A‐C: Labeled QA of CA 1.4 cm at 1‐ (A) and 5‐days (B) post CldU incubation and corresponding data quantification (C). D‐F: Labeled HM of CA 1.4 cm at 1‐ (D) and 5‐days (E) post CldU incubation and corresponding data quantification (F). G‐I: Labeled QA of DC 1.4 cm at 1‐ (G) and 5‐days (H) post CldU incubation and corresponding data quantification (I). J‐L: Labeled QA of DC 1.7 cm at 1‐ (J) and 5‐days (K) post CldU incubation and corresponding data quantification (L). M: quantification of labeling indexes in the PZ of 1.4 cm (TrL) CA QA and HM, and of the 1.4 and 1.7 cm (TrL) DC QA. Error bars represent SD. (***) indicates statistical significance (*P* < 0.05, Wilcoxon rank‐sum test). Abbreviations: HM, hyomandibular; HZ, hypertrophic zone; PZ, proliferative zone; QA, quadrate; RZ, resting zone. Scale bar: 20 μm.

To quantify chondrocyte proliferation parameters in bones undergoing differential growth, 1.4 cm(TrL) CA and DC and 1.7 cm(TrL) DC were incubated in system water containing the nucleotide analog Chloro‐deoxyUridine (CldU) for 6 hours, and left to grow for an additional 24, 48, 72, 96 or 120 hours (chase). CldU‐labelled chondrocytes were then counted in the PZ and the HZ of bones undergoing differential growth: the QA and HM of 1.4 cm(TrL) CA, the QAs of 1.4 cm(TrL) and 1.7 cm(TrL) DC (Figure 5). A similar trend was observed in all GZs examined: CldU+ cells were first detected in the PZ (and the RZ to a lesser degree) alone after a 24 hours chase, and their count progressively decreased in the PZ at subsequent time‐points, while increasing in the HZ (Figure 5A‐L). As expected, the average distance travelled by CldU+ cells into the HZ after a 5d chase paralleled the growth rate of each bone. Doublets of CldU+ cells were generally observed 24 hours after the end of the CldU pulse, suggesting that these cells were in S‐phase during the 6 house pulse and had undergone G2 and M phases 24 house after pulse end. In all QAs, cell number was stable at 1, 2 and 3 days post‐pulse and an increase was observed at 4 days (Figure 5C, F, L), suggesting that an additional round of cell division had taken place. In the CA HM, this increase in CldU+ cell number was observed at 3 days post‐pulse (Figure 5F), suggesting that cell cycle length is shorter in the HM than the QA. Consistent with this idea, the CldU labeling index calculated from 1d chases was significantly higher for the CA HM than for the QAs of CA1.4 cm and DC1.7 cm groups (*P* < .05, Wilcoxon rank‐sum test; Figure 5M). These results suggest that differences in cell cycle length take part in modulating GZ growth rates between anatomical locations, stages and species.

## 3 DISCUSSION

In this study, we investigate endochondral growth and its variation in two species of recently diverged cichlids from Lake Malawi with different adult skull morphologies. By comparing particular GZs in CA and DC across multiple stages, we show that growth rates vary between anatomical locations, stages and species. Differences in chondrocyte proliferation and/or cell enlargement underlie all cases of differential growth, yet their relative contributions vary. Surprisingly, differences in ECM production between GZs do not correlate with different rates of growth. These are the first studies exploring differential bone growth and its underlying cellular causes in ray‐finned fishes.

### 3.1 Diversity of vertebrate endochondral growth strategies

Our previous study of endochondral growth in zebrafish suggested that cell proliferation may be the only process in teleosts that modulates growth rates during postembryonic development.^14^ In this study, we describe differential growth in another teleost taxon (cichlid fishes), and test if variation in cell proliferation similarly underlies growth rate differences, both within and between species. We find differential growth between different GZs and stages within an individual, as well as between species. These cichlid growth rates range from 10.7 μm/day (SY, CA1.6 cm) to 39.3 μm/day (HM, DC1.6 cm), which is an order of magnitude lower than those previously measured in rat GPs, where growth rates range from 47 μm/day (proximal radial GP) to 396 μm/day (proximal tibial GP).^7^

Unlike our results in zebrafish,^14^ we detect significant chondrocyte enlargement in some cichlid GZs and variation in the degree of enlargement between anatomical locations. Enlargement ranges from insignificant in the QA (CA 1.4 cm and DC 1.4 cm) to 3.3‐fold in the HM (CA 1.4 cm). Here again, the degree of enlargement is much lower than that observed in mammalian GPs, where enlargements of 10‐ (rat) to 80‐fold (bat) have been reported.^6^, ^8^

In mammalian long bones, the largest contributor (44%‐59%) to endochondral growth is cell enlargement in the HZ, followed by ECM production in the HZ and PZ (32%‐49%), and cell proliferation (7%‐10%).^7^ In cichlids, our results show that cell proliferation and its variation is the main contributor to QA growth and variation thereof between stages and species, due to either the absence of significant cell enlargement or ECM deposition in the pH zone where bone growth is detected (DC1.4 cm) or the lack of parallel between cell enlargement or ECM deposition and growth rate (DC1.7 cm). However, variation in cell enlargement appears to play important roles in differential growth between the QA and HM bones, as it is only detected in the faster growing HM. It will be interesting to investigate the cellular basis of endochondral growth in other ray‐finned fish species to determine if the presence of cell enlargement (and increased ECM deposition) is limited to certain taxa or is simply present in faster growing bones.

Why would significant chondrocyte enlargement be limited to tetrapods, if indeed it is? A recent study has shown that hypertrophic chondrocytes are the least mechanically stiff cells in the mammalian GP and the authors suggest that secondary ossification centers may have evolved in tetrapods to protect hypertrophic chondrocytes from mechanical loading.^20^ In addition, the degree of chondrocyte enlargement is inversely correlated with cell lifespan in mammals, for example, bat chondrocytes die after 1‐10 hours in the HZ.^8^ Thus, chondrocyte enlargement to facilitate growth may come at the expense of cell survival and bone strength. In contrast, in teleosts where GZ function has been investigated such as zebrafish and cichlids, most cartilage‐derived bones remain in a cartilaginous/perichondral state for weeks before the onset of apoptosis and ossification. Mechanically stiff cartilage may be required during this early period until the bone collar is thick enough to support cartilage‐free bone. Further studies across more species are clearly needed to determine if chondrocyte enlargement is under‐represented in teleosts and its functional significance.

An additional difference between cichlid and tetrapod GZs is the shape of swollen HZ chondrocytes. Cell enlargement is isotropic in the cichlid HM as cells become essentially spherical, while mammalian HZ chondrocyte swelling produces a disproportionate increase in cell height, which in turn produces axial elongation at the GZ.^3^ The lack of anisotropy in cichlid chondrocyte enlargement is likely a result of the low amount of ECM detected in the HM. Do species/bones undergoing greater growth rates produce ECM channels to better direct cell swelling along the axis of growth? Future studies will be necessary to answer this question.

### 3.2 Potential mechanisms of endochondral differential growth in cichlids

What molecular mechanisms mediate differences in cichlid PZ size? Species‐specific differences in PZ height appear at the onset of GZ development in cichlids, suggesting that differential growth reflects differences in embryonic patterning.^17^ In tetrapod long bones, PZ size is maintained by two paracrine factors that interact through a negative feedback loop: *Indian Hedgehog* (*Ihh*) and *Parathyroid Hormone‐Like Hormone* (*Pthlh*).^21^, ^22^ *Ihh* is expressed in prehypertrophic chondrocytes and promotes *Pthlh* expression distally in periarticular chondrocytes. *Pthlh* in turn acts on its receptors in PZ chondrocytes to maintain their proliferation and repress *Ihh* expression.^23^, ^24^ As chondrocytes transition into the pHZ and distance themselves from the *Pthlh* source, they start producing *Ihh*. This feedback loop is largely conserved in cranial base synchondroses, although *Pthlh* is expressed throughout the RZ and PZ.^25^, ^26^ In zebrafish this feedback loop is also initiated at the onset of chondrogenesis in the ceratohyal cartilages, prefiguring the location and presumably the size of the future GZ.^27^ Possible molecular mechanisms mediating PZ size variation in cichlids include changes in *Ihh* and/or *Pthlh* expression levels as well as those of their activators or repressors. *Ihh* expression is promoted by *SMAD1/5*,^28^ while being repressed by *SMAD2/3* ^29^ and by FGF signaling through *Fgfr3*.^30^, ^31^ The regulatory mechanisms mediating GZ‐specific variation in cell‐cycle length have not been investigated, yet the same signals that regulate PZ size perhaps regulate stage‐specific changes as well.

What molecular mechanisms mediate hypertrophic chondrocyte enlargement? In mammals, *Insulin‐like growth factor 1* (*Igf1*) is required for chondrocyte enlargement in the HZ and may play an important role in the establishment of growth‐plate‐dependent cell size.^9^, ^32^, ^33^ The specific expression of *Igf* receptors in particular cichlid growth zones, such as the HM GZ may underlie the cell enlargement found in this specific GZ.

### 3.3 Role of endochondral growth in post‐embryonic ontogeny of the teleost skull

Adult vertebrate morphology is the product of embryonic and postembryonic development, which are often mediated by distinct cellular mechanisms. For example, the skeleton develops in the embryo through the assembly of mesenchymal condensations that prefigure the shape of the embryonic skeleton, while this skeleton will change in size, and sometimes also in shape, through remodeling and localized growth during postembryonic development.^3^, ^34^ Differential growth at GZs is an important mechanism for skeletal shape change, as exemplified by the patent differences in limb proportions between human newborn and adult life stages.

The fish skull is a great subject for the study of differential endochondral growth and its significance on functional morphology. It consists of 20 or more mobile elements that operate during feeding and are modeled as lever systems of various complexities.^35^, ^36^ Lever length, but also the relative positions of levers and axes of rotation influence feeding mechanics. In the particular case of the cichlid skull, the morphological evolution that took place in the three independent adaptive radiations of Lakes Victoria, Malawi and Tanganyika repeatedly followed the same axis of diversification.^37^, ^38^ The opposite extremes defining this axis represent biters (reduced preorbital region) and suction feeders (enlarged preorbital region).^37^ Interestingly, these particular adult morphologies largely appear during postembryonic development in cichlids^16^, ^17^ and in damselfishes,^15^ suggesting that differential growth and remodeling have been under selective pressures during the diversification of these lineages. Cichlids are thus good models to identify the cellular and genetic mechanisms underlying these evolved differences in postembryonic development. In this study, we showed that differential endochondral growth of the QA bone underlies a greater size increase of the preorbital region in DC relative to CA, and that differences in PZ height and perhaps cell‐cycle length underlie this difference in QA growth rate.

## 4 EXPERIMENTAL PROCEDURES

### 4.1 Animal care

All animals were reared and euthanized following protocols approved by the IACUC at the University of North Carolina, Wilmington. DC and CA adults were purchased from pet stores and species were maintained separately at 28°C in 55‐ or 75‐gal tanks. Adults were allowed to breed naturally. Juvenile offspring were collected from females at 10‐15 dpf and transferred to 10‐gal tanks, where they were grown for a week under heavy brine‐shrimp feeding. They were then transferred to 55 gal tanks to maximize growth rate, where they were fed with a mixture of live brine shrimp and dry Zeigler pellets. Adults were fed with Hikari Cichlid Gold medium pellets and 3 mm Northfin Food Cichlid Formula.

### 4.2 Skeletal preparations

All animals were euthanized by immersion in a 168 mg/L tricaine‐S solution (MP Biomedicals).

Adult skeletons (Figure 1): specimens were fixed for 3‐7 days in 10% Normal Buffered Formalin (NBF). Skeletal staining was conducted according to.^39^ Larvae skeletons: 15 dpf larvae, 1 month‐old/1.4 cm (TrL) and 2 month‐old/1.7 cm (TrL) juveniles were fixed for 3 days in 4% paraformaldehyde. Specimens stained with alizarin red and alcian blue as described with 60 mM MgCl_2_.^40^ Live bone staining by calcein green and alizarin red was conducted as described in Kimmel et al., with modifications.^41^ Fish were stained for 24 hours in calcein green, returned to tanks for 14 days, and then chased with alizarin red for 24 hours. Fish were then returned to tanks for 24 hours before fixation overnight in 10% NBF at 4C and stored in 100% ethanol in the dark at 4°C until trypsin digestion as previously described.^41^ Preparations were phenotyped on either a Leica M165FC dissecting microscope or a Zeiss Axioskop 2 FS Plus compound microscope. The dissecting microscope was equipped with a Planapo 1.6X M‐series objective, a Prior L200 fluorescent light source and a Leica DC7000T camera controlled by LAS X software. The compound microscope was equipped with a Qimaging Micropublisher 6 camera controlled by Ocular software.

### 4.3 Histology

Specimens were fixed in Bouin's fixative for 3 hours and rinsed at least 10 times in tap water over several days until rinsed wash did not stain yellow. Individuals were then decalcified for 2 weeks in 20% EDTA, followed by dehydration, clearing and embedding in paraffin wax (Paraplast Plus, Fisherbrand). 10 μm sections were cut and stained with celestine blue b (celestine blue b is a histological stain, it stains nuclei), haematoxylin, alcian blue and direct red as described.^42^ Stained sections were then imaged on a Zeiss Axioskop 2 FS Plus compound microscope equipped with a Qimaging Micropublisher 6 camera controlled by Ocular software.

### 4.4 Cell proliferation assay

Groups of 5‐10 individuals were placed for 6 or 12 hours in a clear box bathing inside their home‐tank and filled with 100 mL of system water containing 100 μg/mL CldU (MP Biosystems). Individuals bathed for 12 hours were then immediately sacrificed and fixed for 3 days in 4% PFA at 4°C. Individuals bathed for 6 hours were returned to their home tank and sacrificed/fixed either 24, 48, 72, 96 or 120 hours later. Specimens were then rinsed three times 10 minutes in PBT and their suspensorium skeletons were dissected out. All subsequent steps were carried out on suspensorium skeletons alone. The following sequence of incubations was used: 1 hour in 10 μg/mL proteinase K (MP Biomedicals) in PBT, 20 minutes in 4% PFA, 3 times 10 minutes in PBSDT (10% Triton‐X, 1% DMSO in PBS), 20 minutes in 2 N HCl, three times 10 minutes in PBSDT, 1 hour in 10% 2‐mercaptoethanol in water, three times 10 minutes in PBSDT, 2 hours in blocking solution (5% NGS in PBSDT), 5 days in 1:100 rat anti‐BrdU antibody (ABCam Ab6326) in PBSDT at 4°C, six times 1 hour in PBSDT, 2 days in 1:400 goat anti‐rat‐HRP antibody (Jackson Immuno Research, NC9845108) in PBSDT at 4°C, five times 1 hour in PBSDT. Signal development was achieved with Pierce DAB Substrate Kit (Thermo Scientific 34002) following manufacturer's instructions while doubling recommended DAB solution volume. Once signal was detected, samples were rinse two times 10 minutes in PBT and kept in 35% Glycerol in PBT before mounting between slide and coverslip for microscope observations and imaging.

### 4.5 In situ hybridization and immunostaining

For cryosections, larvae were processed as described.^43^ Larvae were cut as 16 μm sections on a Leica CSM1816 cryostat. For in situ hybridization (ISH), sections were hybridized as described.^43^ ISH probes were generated from RT‐PCR amplified regions of CA *col2a1a* and *col10a1a* cDNAs using the following primers designed from published *Maylandia zebra* sequences: CA_col2a1aF: CTCAAGGCAAAGTTGGACCT, CA_col2a1aR: GACTCTCCTTTCTGTCCACG, CA_col10a1aF: GCAAGAGGATTTCAGGGTGA, CA_col10a1aR: GGCAATCAAGAACCCAGAGA. The amplified CA *col2a1a* fragment was 868 bp long and over 99% identical to its *M. zebra* ortholog (Malawi cichlid reference genome, sequence ID XM_00454229.4). The amplified CA *col10a1a* fragment was 972 bp long and over 99% identical to its *M. zebra* ortholog (Malawi cichlid reference genome, sequence ID XM_004566213.3). Immunohistochemistry was performed as described 62 without the proteinase K step, with mouse anti‐PCNA (Proliferating Cell Nuclear Antigen; 1/500; Santa Cruz Biotech; PC10), Alexa Fluor 488 conjugated Donkey anti‐mouse secondary antibody (1/100 Jackson Immunoresearch 715‐545‐150) and DAPI (1:2000 Life Technologies D1306). Fluorescence imaging was conducted on a Leica SP8 confocal microscope with a 63× oil immersion objective.

### 4.6 Data collection and analysis

Cell cross‐sectional area and height measurements were collected in FIJI from images captured under Nomarski illumination with a 63x water‐immersion objective on a Zeiss Axioskop 2 FS plus microscope. Cell contours in regions of interest were manually drawn using the freehand selection tool to obtain individual cell cross‐sectional areas and cell dimensions parallel to the direction of endochondral growth. Cell cross‐sectional area and height averages and standard deviations were calculated in Microsoft Excel, and graphed in R using ggplot2 package. To determine ECM percentage of total skeletal area for each image, the sum of all cell cross‐sectional areas was subtracted from the total area of the region of interest. Statistical significance was determined using Tukey's test of Honest Significant Difference in R.

## AUTHOR CONTRIBUTIONS

**Savannah Leigh Johnson:** Data curation (equal); investigation (equal). **Brian Heubel:** Investigation (equal); visualization (equal); writing – review and editing (supporting). **Carson Bredesen:** Investigation (equal). **Thomas Schilling:** Writing – review and editing (equal). **Pierre Le Pabic:** Conceptualization (lead); data curation (equal); formal analysis (equal); funding acquisition (equal); investigation (equal); methodology (equal); project administration (lead); writing – original draft (lead); writing – review and editing (equal).
